# Cachexia Prevalence in a Population of Moroccan Women with Rheumatoid Arthritis

**DOI:** 10.31138/mjr.250823.cpp

**Published:** 2023-08-25

**Authors:** Hamza Toufik, Najlae El Ouardi, Mohamed Ahmed Ghassem, Julien H. Djossou, Laila Taoubane, Abderrahim Majjad, Abdellah El Maghraoui, Ahmed Bezza

**Affiliations:** 1 Rheumatology Department, Military Hospital Mohammed V, Rabat, Morocco,; 2 Cabinet of Rheumatology, Rabat, Morocco

**Keywords:** Rheumatoid arthritis, body composition, rheumatoid cachexia, fat mass, fat-free mass, dual-energy x-ray absorptiometry

## Abstract

**Objective:**

To assess body composition in women with rheumatoid arthritis (RA) compared to healthy controls, to calculate the prevalence of rheumatoid Cachexia (RC), and to identify the associated factors.

**Methods:**

We conducted a case-control study on 112 female patients with RA according to the 2010 American College of Rheumatology/European League Against Rheumatism classification criteria for RA; and 224 age-matched healthy women. Body composition (BC) and bone mineral density (BMD) scans were obtained using Dual-energy X-ray absorptiometry (DXA). RC was defined by a fat-free mass index (FFMI) below the 10^th^ percentile and a fat mass index (FMI) above the 25^th^ percentile compared with the control group. We conducted a comparison between RA patients and healthy controls then a multiple regression analysis was conducted where the dependant variable is the presence of RC.

**Results:**

RC prevalence was 42.85% while the mean body mass index (BMI) was the same in both groups. RA patients had a higher FM and lower FFM comparing to healthy controls. In our population, 78.60% of patients were on methotrexate and 12.50% on anti TNF therapy. Comparison between patients with and without RC showed that patients with RC have a higher proportion of erosive arthritis and of active disease. Regression logistic analysis showed that RC was significantly associated to erosive arthritis and active disease (OR at 33.31 (8.42-131.70) and 8.98 (1.64-49.20) respectively), independently of age, erythrocyte sedimentation rate, C-reactive protein, disease duration, steroid cumulative dose and biologic Disease-Modifying Anti-Rheumatic Drugs(bDMARDs) use.

**Conclusion:**

Our study showed that almost half of our RA patients have RC, even with a high BMI.

## INTRODUCTION

Rheumatoid arthritis (RA) is a chronic systemic inflammatory disease that causes an increasing disability and cardiovascular mortality.^1^ Interestingly, both of these complications are associated with a change in body composition.^2^

The term “body composition” (BC) refers to the quantification of the different compartments of the human body, thus fat mass (FM) and fat-free mass (FFM), the latter includes body water, bones, and organs, but primarily, muscles.^3^ The altered body composition of inflammatory rheumatic diseases was first identified in 1873 by Sir James Paget and given the name rheumatoid cachexia (RC).^4^ RC is different from classic cachexia because it is characterized by a decrease in FFM and a maintenance or increase in FM with little or no changes of Body Mass Index (BMI).^5^ RC contributes to physical impairment in patients with RA due to the decrease in muscle mass and force and may increase the risk of cardiovascular morbi-mortality as a consequence of fat mass increase.^6,7^ Studies have shown that clinical outcomes also include increased risk of infections.^8^ Thus, the clinical benefits of RC evaluation could be multiple. On one hand, RC might somehow reflect the functional impact of RA, giving early insight into impaired physical activity and risk of falls. And on the other hand, a cardiovascular and metabolic assessment of RA can go through that of RC. Otherwise, anti-TNFα seems to be a good indication in RA with RC and has shown some benefit.^9^

Body Mass Index (BMI) is widely used to assess nutritional status (obesity, normal and lean), but does not give an estimation on the quantity nor distribution of fat mass.^10,11^ Dual-energy X-ray absorptiometry (DXA) is a non-invasive, low-radiating, reproducible and rapid technique, initially, is considered the gold standard for measuring bone mineral density and is currently validated as a measurement tool for body composition in patients with RA.^12,13^

The prevalence of RC is variable and depends directly on its definition, populations, and methods used for body composition assessment.^10,11,14^ According to a recent meta-analysis, the prevalence of RC is 15-32%.^15^ We have already assessed body composition using as a reference a large Swiss population of healthy adult subjects,^16,17^ but without a control group, as in most previous studies.^10,18,19^ In the present study, we used as reference a Moroccan database and with a healthy control group. This research study aims to investigate the prevalence of cachexia in a population of Moroccan women with RA. By conducting this study, we can identify the extent of cachexia in Moroccan women with RA, which will help to improve the understanding of the disease and its management. Additionally, the study will provide valuable information for healthcare professionals in Morocco to develop targeted interventions that aim to prevent or manage cachexia in RA patients.

## METHODS

### Patients and healthy controls

We conducted a case-control study at the Rheumatology Department of the Military Hospital of Rabat, Morocco, on 112 women with RA, according to the 2010 American College of Rheumatology/European League Against Rheumatism classification criteria for RA. The control group consisted of 224 age-matched healthy women with two controls for one case. This group was extracted from a healthy general population (database of the study of body composition by DXA in a Moroccan female population aged 20 to 80 years, data submitted for publication). The subjects signed an informed consent and the study was approved by our local ethics committee (Military Hospital Mohammed V, Rabat) and was performed in accordance with the Helsinki declaration. Exclusion criteria were the presence of another inflammatory rheumatic disease, chronic infections, chronic renal or liver diseases, and cancer. Demographic characteristics and clinical examination, comprising disease duration was defined as the time elapsed between the onset of first disease-related symptoms and diagnosis, swollen joint count (28 joints) and tender joint count (28 joints), were collected. The Disease Activity Score at 28 joints-ESR(DAS28-ESR) was measured. An active disease was defined as DAS28-ESR> 5.1. Radiological status was assessed through the clinical file and patients were classified as RA with or without erosive arthritis. The use of conventional synthetic DMARDs (csDMARDs), bDMARDs and steroids was collected.

### Body composition assessments by DXA

Body composition was measured with total body DXA using the same machine. The whole-body scan used the DXA system’s automated software, which provided compositional estimation of legs, arms, trunk, head, and the whole body. Scans were performed with the subject wearing light indoor clothing and with no detachable metal objects present. The precision of soft tissue analysis for a Lunar Prodigy is 1% for FFM and 2% for FM. FFM and FM were expressed in absolute kilogram, and FM also as percentage of total mass. The normal reference value for FM percent is 20% to 30% for women and 12% to 20% for men. FFM index (FFMI; kg/m^2^) and FM index (FMI; kg/m^2^) were also calculated. To define RC, we adapted the definition by Engvall et al(16), who classified the patients as RC if FFMI was below the 10th percentile and FMI above the 25th percentile of the normal population using our Moroccan reference database values. This definition was used in most of the studies with good quality focusing on this subject. In a recent meta-analysis (15) 6 out of 8 included studies used this definition.

### Bone mineral density measurements

The bone mineral density (BMD) of the lumbar spine, total hip and femoral neck was measured using DXA (Lunar Prodigy, General Electric, Madison, WI, USA) in all patients and controls. All BMD measurements were carried out by 2 experienced technicians. The coefficient of variation of the phantom precision was 0.08 and the reproducibility assessment in clinical practice showed in a previous study a smallest detectable difference of 0.04 g/cm^2^ (spine) and 0.02 (hips). The World Health Organization (WHO) classification system was applied, defining osteoporosis as T score ≤ -2.5 and osteopenia as -2.5 < T score < -1. Study participants were categorized by the lowest T score of the L1-L4 lumbar spine, femur neck, or total femur.

### Statistical analysis

Quantitative variables are expressed as mean ± standard deviation (SD) or median (interquartile range), depending on the normality or not of the distribution. Frequency and percentage were used for qualitative variables. Comparisons between RA patients and healthy controls were performed using the Student’s t-test or Mann-Whitney U test, depending on the distribution of quantitative variables and the Chi-square test for qualitative variables. A Multiple regression analysis was conducted where the dependant variable was the presence of RC and the independent variables the potential risk factors. Statistical significance was set at p <0.05. Analyses were performed with SPSS Version 23.0 (SPSS, Chicago, Illinois, USA).

## RESULTS

### Subject characteristics

Characteristics of 112 RA patients and 224 controls are presented in **Table 1**. The mean ± SD of the weights and BMI were similar between the two groups. The FFM was lower in RA patients compared with controls (p<0.01). The median (IQR) for disease duration and mean± SD for DAS 28-ESR score were 7(3-12) years and 4.46 ± 1.70 respectively. Among RA patients 68.75% and 69.64% were positive for RF (Rheumatoid Factor) and ACPA (Anticyclic Citrullinated Peptide Antibodies) respectively. Using our definition, RC was observed in 48 patients (42.85%). The used drugs were methotrexate in 78.60% of cases, rituximab in 32.14%, sulfasalazine in 13.40%, and anti TNF therapy in 12.50% of cases.

**Table 1.** Characteristics of rheumatoid arthritis patients and controls.

	**RA patients N=112**	**Controls N=224**	**p**
Age (years), m (SD)	54.21 (11.11)	55.15 (10.56)	ns
Weight (kg), m (SD)	71.08 (11.56)	73.25(11.73)	ns
Height (m), m (SD)	1.58 (0.06)	1.57 (0.06)	ns
BMI (kg/m^2^), m (SD)	28.70 (4.56)	29.83(4.78)	0.04
FM (kg), m (SD)	38.51 (7.01)	36.77 (4.55)	0.01
FFM (kg), m (SD)	30.43 (9.59)	34.17 (8.28)	<0.01
FFMI (kg/m^2^), m (SD)	11.93 (3.86)	13.93(3.43)	<0.01
FMI (kg/m^2^), m (SD)	14.92 (1.83)	14.97 (1.83)	ns
% FM, mean (SD)	42.85 (8.86)	47.44 (5.59)	<0.01
LS BMD (g/cm^2^), m (SD)	0.98 (0.20)	1.05 (0.21)	0.01
TH BMD (g/cm^2^), m (SD)	0.89 (0.14)	0.94 (0.16)	0.01
Disease duration (years), median (IQR)	7.00 (3.00-12.00)		
RF-positive, n (%)	77 (68.75)		
Anti-CCP–positive, n (%)	78 (69.64)		
Erosive arthritis, n (%)	59 (52.67)		
Rheumatoid Cachexia, n (%)	48 (42.85)		
DAS 28-ESR, m (SD)	4.46 (1.70)		
Active disease, n (%)	80 (71.42)		
ESR (mm/h), m (SD)	30.83(22.62)		
CRP (mg/l), m (SD)	18.48 (24.68)		
Medication			
– Steroid cumulative dose (g), median (IQR)	14.14 (5.40-27.37)		
– csDMARDs			
○ Methotrexate, n (%)	88 (78.60)		
○ Sulfasalazine, n (%)	15 (13.40)		
○ Leflunomide, n (%)	5 (4.50)		
– bDMARDs			
○ Anti TNF therapy, n (%)	14 (12.50)		
○ Rituximab, n (%)	36 (32.14)		

anti-CCP: anticyclic citrullinated peptide antibodies; bDMARDs: biologic Disease-Modifying Anti-Rheumatic Drugs; BMD: bone mineral density; csDMARDs: conventional synthetic Disease-Modifying Anti-Rheumatic Drugs; CRP: C-reactive protein; DAS28: 28-joint Disease Activity Score; ESR: erythrocyte sedimentation rate; FFM: free-fat mass; FFMI: fat-free mass index; FM: fat mass; FMI: fat mass index; IQR: interquartile range; LS: lumbar spine; m (SD): mean (standard deviation); n/N: number; NS: not significant; RF: rheumatoid factor; TH: total hip.

### Rheumatoid Cachexia (RC)

**Figure 1.**
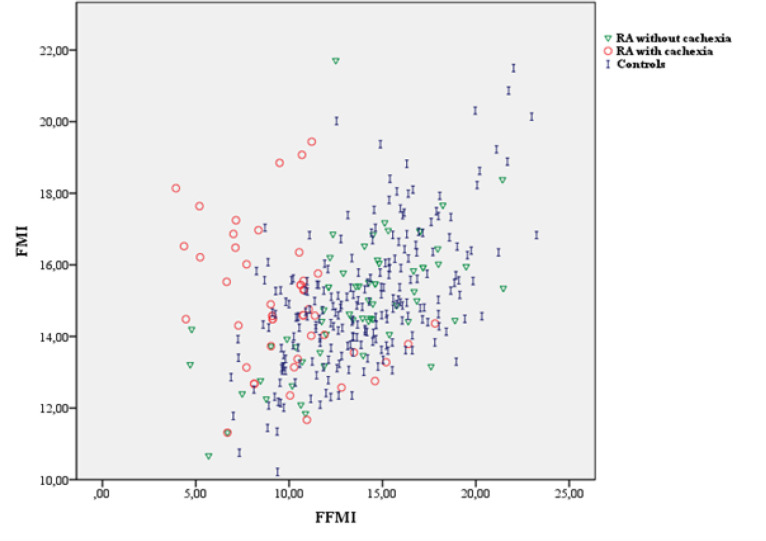
Distribution of FFMI and FMI relative to RA with or without cachexia and controls.

**Table 2** shows the comparison between patients with and without RC. The mean age of patients with RA with cachexia was 53.17 ±10.38 years. More RA patients with RC had an active disease (85.41 % vs. 60.93%) and an erosive disease (91.66% vs. 23.43%). No differences were found between the two groups with regard to symptomatic disease severity parameters. **Figure 1** shows the distribution of FFMI and FMI relative to RA with or without cachexia and controls.

**Table 2.** Comparison between patients with and without rheumatoid cachexia.

	**Patients without cachexia N=64**	**Patients with cachexia N=48**	***p***
Age (years), m (SD)	55.02 (11.72)	53.17 (10.38)	ns
Weight (kg), m (SD)	73.63 (11.37)	67.32 (10.85)	<0.01
Height (m), m (SD)	1.57 (0.05)	1.58 (0.07)	ns
BMI (kg/m^2^), m (SD)	29.74 (4.54)	27.14 (4.13)	<0.01
FM (kg), m (SD)	37.60 (5.44)	39.82 (8.65)	ns
FFM (kg), m (SD)	34.19 (9.26)	25.36 (7.65)	<0.01
% FM (%), m (SD)	45.89 (7.80)	38.71 (8.69)	<0.01
FMI (kg/m^2^), m (SD)	13.60 (3.63)	9.69 (2.98)	<0.01
FFMI (kg/m^2^), m (SD)	14.90 (1.80)	14.97 (1.89)	ns
LS BMD (g/cm^2^), m (SD)	0.98 (0.21)	0.98 (0.19)	ns
TH BMD (g/cm^2^), m (SD)	0.90 (0.15)	0.88 (0.13)	ns
Disease duration (years), m (SD)	9.63 (8.75)	8.02 (5.71)	ns
Erosive arthritis, n (%)	15 (23.43)	44 (91.66)	<0.01
Active disease (DAS 28-ESR>5.1), n (%)	39 (60.93)	41 (85.41)	<0.01
DAS 28-ESR, m (SD)	4.23 (1.79)	4.74 (1.57)	ns
ESR (mm/h), m (SD)	29.27 (20.28)	32.80 (25.67)	ns
CRP (mg/l), m (SD)	15.61 (17.44)	22.35 (32.18)	ns
RF-positive, n (%)	45 (70.31)	31 (65.95)	ns
Anti-CCP–positive, n (%)	47 (73.43)	29 (60.41)	ns
Medication			
– Steroid cumulative dose (g), m (SD)	20.43 (24.17)	21.85 (22.99)	ns
– csDMARDs			
○ Methotrexate, n (%)	50 (78.12)	38 (79.16)	ns
○ Sulfasalazine, n (%)	10 (15.62)	5 (10.41)	ns
○ Leflunomide, n (%)	3 (4.68)	2 (4.16)	ns
– bDMARDs			
○ Anti TNF therapy, n (%)	8 (12.50)	6 (12.50)	ns
○ Rituximab, n (%)	21(32.81)	15 (31.25)	ns

anti-CCP: anticyclic citrullinated peptide antibodies; bDMARDs: biologic Disease-Modifying Anti-Rheumatic Drugs; BMD: bone mineral density; csDMARDs: conventional synthetic Disease-Modifying Anti-Rheumatic Drugs; CRP: C-reactive protein; DAS28: 28-joint Disease Activity Score; ESR: erythrocyte sedimentation rate; FFM: free-fat mass; FFMI: fat-free mass index; FM: fat mass; FMI: fat mass index; IQR: interquartile range; LS: lumbar spine; m (SD): mean (standard deviation); n/N: number; NS: not significant; RF: rheumatoid factor; TH: total hip.

**Table 3** shows a multiple regression analysis where RC was the dependent variable: erosive arthritis and active disease were significantly associated to RC with an OR at 33.31 (8.42-131.70) and 8.98 (1.64-49.20) respectively, independently of age, Erythrocyte Sedimentation Rate, C-reactive protein, disease duration, steroid cumulative dose and bDMARDs (Anti TNF and Rituximab).

**Table 3.** Multiple regression analysis with the presence of rheumatoid cachexia as the dependent variable.

	**OR**	**95% confidence interval**	***p***
Age	1	0.95-1.06	ns
Erosive arthritis	33.31	8.42-131.7	<0.01
Active disease (DAS 28-ESR>5.1)	8.98	1.64-49.20	<0.05
ESR	0.99	0.97-1.02	ns
CRP	1.006	0.97-1.03	ns
Disease duration	0.96	0.86-1.06	ns
Steroid cumulative dose	1		ns
bDMARDs (Anti TNF and Rituximab)	0.7	0.53-1.12	ns

bDMARDs: Biologic disease-modifying anti-rheumatic drugs; CRP: C-reactive protein; DAS28-ESR: 28-joint Disease Activity Score; ESR: erythrocyte sedimentation rate; NS: not significant; OR: Odds Ratio.

## DISCUSSION

The main result of the present study is that RA patients have higher FM and lower FFM compared to healthy controls while being considered overweight according to the BMI. This makes BMI an inappropriate indicator of RC in RA.

Several studies showed that anthropometric parameters including BMI do not reflect RC.^20-22^ In a large population of RA patients, mainly women, Wolfe and Michaud showed that BMI was not a reliable tool to detect the malnutrition in RA patients.^20^ In a cross-sectional study, 44% of women with a normal BMI had low FFMI, while 40% of women and 75% of men in the normal BMI category had high or very high FMI.^22^ However, the precision and the availability make DXA a useful and convenient tool for body composition assessment.^13^

In our study, we used DXA and the definition proposed by Engvall et al. using a healthy Moroccan population as a reference.^19^ We found RC in 42.85% of RA patients. This prevalence is similar to that reported in a previous study^17^ which used a reference population from Switzerland.^16^ Using the same definition and evaluation method, Hugo et al.^18^ and Elkan et al.^6^ found RC prevalence of 18% while Engvall et al.^19^ found a prevalence of 38%. Lombard et al. found RC prevalence of 10.3% using the same definition but the anthropometric measurements to assess RC in RA patients.^23^ Using Bioelectrical Impedance Vector, C. S-Díaz et al. found RC prevalence of 21% in RA patients.^24^ The prevalence of RC is variable and depends directly on its definition, populations, and methods used for body composition assessment.^15^ We found a high prevalence compared to the studies that used the same definition and the same evaluation method. This could be explained, in part, by the high frequency of active RA in our population and the long duration of the disease compared to other studies.^6,18,19^

In our study, this prevalence significantly associated with disease activity and erosive arthritis. Association between RC and disease activity is controversial, despite that some basic sciences provide arguments that suggest that body composition changes can modulate the clinical status of RA.^25^ A systematic review did not provide enough data to assess the relationship between body composition and clinical activity.^26^

The mechanisms causing RC are not completely understood, but muscle mass loss is likely related to pro-inflammatory cytokines such as tumour necrosis factor-α (TNF-α), interleukin 1 (IL-1) and IL-6 (25,27). TNFα (formerly called cachectin) is a pivotal cytokine in RA pathogenesis. It increases the whole-body protein catabolism and leads to a wasting of FFM and an increase in FM (28). In our study, 78.60% of patients were on methotrexate and 12.50% were on anti TNF therapy without statistically significant difference between patients with and without RC. However, little evidence shows that anti TNF therapy is effective in cachexia in humans. Lemmey et al. showed that treat-to-target (T2T), despite its enhanced efficacy in reducing disease activity and joint damage, has not improved patients’ body composition relative to previous treatment regimens.^29^ In the Veterans Affairs Rheumatoid Arthritis (VARA) Registry, methotrexate use was associated with a lower risk of RC whereas the use of steroidal anti TNF therapy was not associated with a change in BC.^30^ Metsios et al. showed that after 12 weeks of anti TNF therapy, there were significant improvements in disease activity and physical function, as well as physical activity and protein intake, but no significant changes in FFM.^31^ Marcora et al. showed that anti TNF therapy with etanercept is not superior to that with methotrexate for the treatment of rheumatoid cachexia over a period of 24 weeks in patients with early RA.^32^ The main limitation of our study was that we did not assess physical activity and nutritional intake of the study population. However, our study used a significant cohort of healthy women as a matched control group and therefore strengthening the validity of our results.

## CONCLUSION

We conclude that almost half of our RA patients have RC, even with a high BMI. Therefore, our study suggests that further attention to the assessment of BC in women with RA is warranted. Although DXA is currently a validated tool for BC assessment, further studies are needed to better define RC and assess its long-term consequences and the sensitivity to change of BC parameters with the current treatments used to manage RA.
